# Assessment of Use of *ICD-9* and *ICD-10* Codes for Social Determinants of Health in the US, 2011-2021

**DOI:** 10.1001/jamanetworkopen.2023.12538

**Published:** 2023-05-09

**Authors:** Amil R. Agarwal, Laura Prichett, Amit Jain, Uma Srikumaran

**Affiliations:** 1 Department of Orthopedic Surgery, Johns Hopkins Medicine, Baltimore, Maryland; 2 Department of Orthopaedic Surgery, George Washington University School of Medicine and Health Sciences, Washington, DC; 3 Biostatistics, Epidemiology, and Data Management, Johns Hopkins Medicine, Baltimore, Maryland

## Abstract

This cross-sectional study examines trends in the use of *International Classification of Diseases, Ninth Revision (ICD-9)* and *International Statistical Classification of Diseases and Related Health Problems, Tenth Revision (ICD-10)* codes for social determinants of health in the US from 2011 to 2021.

## Introduction

Prior literature suggests that social determinants of health (SDOH) account for approximately 80% of health outcomes in the US.^1^ Although addressing SDOH has demonstrated improvements in population health at the hospital, payer, and practitioner levels, the current fee-for-service payment systems do not incentivize these efforts. The *International Classification of Diseases, Ninth Revision (ICD-9)* and *International Statistical Classification of Diseases and Related Health Problems, Tenth Revision (ICD-10)* diagnosis codes for SDOH provide a means to comprehensively document SDOH variables, but they have been underused historically.^2^ This study aimed to observe the trends in use of SDOH codes in the US from 2011 to 2021.

## Methods

A cross-sectional study was conducted using patient data from January 2011 to December 2021 from the Mariner data set of the PearlDiver database. This data set contains information from varying payer types for more than 150 million patients (eAppendix in Supplement 1). This study followed the Strengthening the Reporting of Observational Studies in Epidemiology (STROBE) reporting guidelines for cross-sectional studies and was approved by the George Washington University institutional review board. Because this data set contains access to retrospective, deidentified aggregate patient information, informed consent of patients was waived in accordance with 45 CFR §46. This study was performed in accordance with the ethical standards of the 1964 Declaration of Helsinki^3^ and its later amendments or comparable ethical standards.

Patients with at least 1 *ICD-9* or *ICD-10 *code for adverse SDOH were observed (eTable in Supplement 1). These codes were stratified according to 5 identified domains: education, health care, environmental, social, and economic.^4^ The change in percentage of patients with at least 1 SDOH and the different domains were viewed from 2011 to 2021. Linear regression analysis was conducted to observe statistically significant temporal changes in the use of SDOH codes, with a significance threshold of *P* < .05. SE was reported to show variation. Analysis was conducted using R Statistical software version 4.1.3 (R Project for Statistical Computing) provided by the PearlDiver database. Data analysis was conducted from November 2022 to January 2023.

## Results

From 2011 to 2021, all 156 486 859 patients in the database were queried, and 8 712 741 (5.6%) had at least 1 adverse SDOH code. From 2011 to 2021, the use of at least 1 SDOH code increased from 0.4% (400 888 patients) to 6.8% (7 094 579 patients) (difference, 6.4%; SE, 1.15%; *P* < .001) (Figure), with the largest increase occurring between 2014 to 2016. Among the domains, the use of economic *ICD-9* and *ICD-10* codes increased the greatest from 0.1% (56 039 patients) to 6.3% (6 526 094 patients) (difference, 6.2%; SE, 1.18%; *P* < .001) (Figure). The use of *ICD-9* and *ICD-10* codes by domain increased as follows: from 0.0% (24 839 patients) to 0.2% (156 846 patients) for educational (difference, 0.2%; SE, 0.02%; *P* < .001), from 0.2% (160 524 patients) to 0.7% (725 218 patients) for societal (difference, 0.5%; SE, 0.04%; *P* < .001), and from 0.00% (1096 patients) to 0.01% (6011 patients) for health care (difference, 0.01%; SE, 0.00%; *P* < .001) (Figure). Finally, there was no significant increase in the use of environmental *ICD-9* and* ICD-10* codes (Figure).

**Figure.**
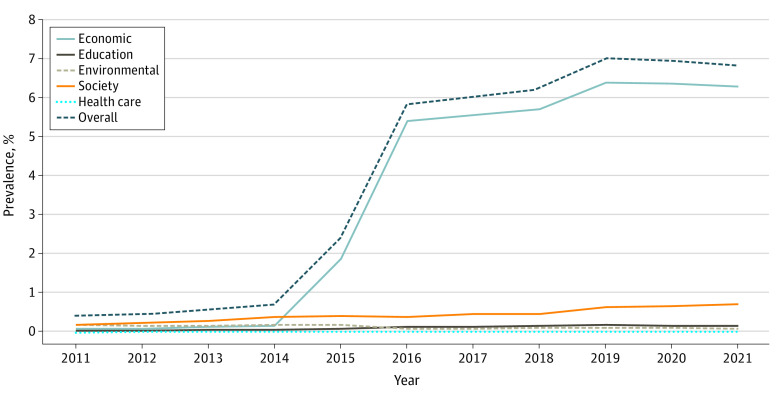
Change in Use of *International Classification of Diseases, Ninth Revision* and *International Statistical Classification of Diseases and Related Health Problems, Tenth Revision* Codes for Social Determinants of Health in the US, 2011-2021 Graph shows change in code use overall and for the social determinants of health domains of economic, education, environmental, society, and health care.

## Discussion

This cross-sectional study found that from 2011 to 2021, there was an increase in the use of *ICD-9* and *ICD-10* codes for SDOH. The largest increase, from 2014 to 2016, most likely represents the transition in October 2015 to *ICD-10*, which incorporates more SDOH codes than *ICD-9* did (eTable in Supplement 1). Other explanations include but are not limited to the transition from local to large vendor electronic health care records, the change from a fee-for-service to a value-based care system following the Patient Protection and Affordable Care Act in 2010, initiatives stressing the importance of proper coding, and the rapid incorporation of Medicare Advantage plans.^5,6^ Despite this increase in use, we still suspect that the total population experiencing adverse SDOH is underrepresented. However, this study is limited in its inability to quantifiably determine how underused these SDOH codes are because of a lack of highly used SDOH surrogates.

To reach a representative use rate, other initiatives, namely financial ones, need to be properly incorporated into practices. Medical comorbidities and other health care factors are widely used in risk adjustment models, but SDOH are not. Because SDOH are associated with more than 80% of health outcomes,^1^ we hypothesize that widespread and accurate incorporation of these codes in all risk adjustment models would lead to both an increase in use of these codes and improvements in established models for risk adjustment.

Unfortunately, recording adverse social factors has been associated with difficulties in access to affordable health care, thereby worsening inequities.^1^ Therefore, widespread incorporation of these codes needs to be coupled with initiatives that prevent the introduction of disparities to care.

## Notes

Supplement 1. **eAppendix.** Supplemental Methods **eTable.** Adverse Social Determinant of Health (SDOH) *International Classification of Diseases, Ninth Revision (ICD-9)* and *International Statistical Classification of Diseases and Related Health Problems, Tenth Revision (ICD-10)* Diagnosis Codes Stratified by Their 5 Domains

Supplement 2.
Data Sharing Statement
