# Supplementary material for: Assessment of Use of ICD-9 and ICD-10 Codes for Social Determinants of Health in the US, 2011-2021
Source: JAMA Netw Open. 2023 May 9;6(5):e2312538. doi: 10.1001/jamanetworkopen.2023.12538 (PMC10170331; doi:10.1001/jamanetworkopen.2023.12538)
Supplement: Supplement 1. — eAppendix. Supplemental Methods eTable. Adverse Social Determinant of Health (SDOH) International Classification of Diseases, Ninth Revision (ICD-9) and International Statistical Classification of Diseases and Related Health Problems, Tenth Revision (ICD-10) Diagnosis Codes Stratified by Their 5 Domains [file jamanetwopen-e2312538-s001.pdf]

Supplemental Online Content

Agarwal AR, Pritchett L, Jain A, Srikumaran U. Assessment of use of *ICD-9* and *ICD-10* codes for social determinants of health in the US, 2011-2021. *JAMA Netw Open*. 2023;6(5):e2312538. doi:10.1001/jamanetworkopen.2023.12538

eAppendix. Supplemental Methods
eTable. Adverse Social Determinant of Health (SDOH) *International Classification of Diseases, Ninth Revision (ICD-9)* and *International Statistical Classification of Diseases and Related Health Problems, Tenth Revision (ICD-10)* Diagnosis Codes Stratified by Their 5 Domains

This supplemental material has been provided by the authors to give readers additional information about their work.

© 2023 Agarwal AR et al. *JAMA Network Open.*

eAppendix. Supplemental Methods

Study Design

A retrospective cross-sectional analysis of the utilization of adverse Social Determinant of Health (SDOH) International Classification of Disease (ICD) codes was conducted using the Mariner Dataset of the PearlDiver database. This a descriptive, observational study to observe whether and to what degree the utilization of these ICD codes has changed throughout the period of this national, insurance claims database (January 2011 to December 2021). First the overall, utilization rate of at least one of these adverse SDOH ICD 9/10 diagnosis codes was observed. Next, the utilization of these codes was observed based on the five defined domains for adverse SDOH (education, healthcare, environmental, social, and economic) [3].

Database

The Mariner dataset of the PearlDiver database contains patient all-payers claims information of 150 million patients observed from 2011 to 2021. For each given year, there are approximately 100 million patients, representative of approximately 30% of the United States population. As we only have access to patient claims information from 2011 to 2021, only these periods were observed. The dataset includes reporting of both private and publicly insured individuals. In total, the dataset includes 156,486,859 patients. Regarding sex, 56% were women and 44% were men. Regarding age, 26% were less than 18, 9% were between 18-24, 9% were between 25-34, 22% were between 35-54, 13% were between 55-64, and 21% were 65 years and older. Regarding insurance, 25% were enrolled in Medicaid, 14% were enrolled in Medicare, and the rest were enrolled in non-Medicare/Medicaid plans. Regarding geographic region, 21% were in the Midwest, 20% were in the Northeast, 40% were in the South, and 19% were in the West. The dataset is accessible to paid users of the PearlDiver database. Unlike other insurance claims

© 2023 Agarwal AR et al. *JAMA Network Open.*

databases, PearlDiver does not permit access to individual records but uses an interface that permits users to observe aggregate data and conduct statistical analysis of the data within the interface. All statistical analysis from the server is conducted using R Software. The utilization of this dataset was deemed approved by our local Institutional Review Board. As this contains access to retrospective, de-identified aggregate patient information, informed consent of patients was waived.

Outcomes

The primary outcome was the overall utilization of at least one adverse SDOH ICD 9/10 code. All ICD 9/10 SDOH codes can be found in the eTable. Based on prior studies and the United States Healthy People 2030 Initiative, these codes can be stratified into five domains: education, healthcare, environmental, social, and economic. Education adverse SDOH include early childhood development problems as well as inadequate education or literacy. Healthcare adverse SDOH include inaccessibility, unavailability, or other issues with healthcare or medical facilities. Environmental adverse SDOH include lead or mold exposure, disaster exposure, and safety problems (history of sexual, physical, or psychological abuse/assault). Social adverse SDOH include incarceration as well as legal/psychosocial issues. Lastly, economic adverse SDOH include unemployment, unsafe occupation environment, food insecurity, housing instability, and financial hardship. The secondary outcome for this study was the utilization of these adverse SDOH based on these five domains.

Statistical Methods

This was a descriptive study, and thus limited statistical analysis was conducted. However, to observe statistically significant differences in the utilization of SDOH ICD codes overtime, linear regression analysis was conducted for the overall adverse SDOH utilization as

© 2023 Agarwal AR et al. *JAMA Network Open.*

well as for the utilization of the five adverse SDOH domains. A p-values of less than 0.05 was considered statistically significant. All statistical analysis was conducted in R Software, provided by the PearlDiver database.

© 2023 Agarwal AR et al. *JAMA Network Open.*

eTable. Adverse Social Determinant of Health (SDOH) *International Classification of Diseases, Ninth Revision (ICD-9)* and *International Statistical Classification of Diseases and Related Health Problems, Tenth Revision (ICD-10)* Diagnosis Codes Stratified by Their 5 Domains

	***ICD-9* Codes**	***ICD-10* Codes**
Economic	V600, V601, V6089, V609, V605, V620, V621, V6229, V713, 9942, 9943, 99552, 99584, V602, V691, E9041, E9042,	Z724, Z590, Z591, Z594, Z595, Z596, Z597, Z598, Z599, Z91120, Z560, Z5689, Z569, Z563, Z562, Z561, Z564, Z565, Z566, Z5681, Z570, Z571, Z572, Z5731, Z5739, Z574, Z575, Z576, Z577, Z578, Z579, T730XXD, T730XXS, T730XXA, T731XXA, T731XXD, T731XXS, T738XXA, T738XXD, T738XXS, T739XXA, T739XXD, T739XXS, X58XXXA, X58XXXD, X58XXXS, E630, E631, E638, E639, E40, E41, E42, E43, E440, E441, E45, E46, E5111, E5112, E519, E52, E530, E531, E538, E539, E54, E550, E559, E560, E561, E568, E569, E58, E59, E60, E610, E611, E612, E613, E614, E615, E616, E617, E618, E619, E640, E641, E642, E643, E648, E649,
Education	V6129, V6121, V623, 99550, 99551, 99553, 99554, 99555, 99559,	Z62898, Z629, Z620, Z6221, Z6222, Z6229, Z62810, Z62811, Z62812, Z62813, Z62819, Z62820, Z62821, Z62822, Z62890, Z62891, Z552, Z553, Z554, Z558, Z559, Z69010, Z734, T7402XA, T7402XD, T7402XS, T7412XA, T7412XD, T7412XS, T7422XD, T7422XS, T7422XA, T7432XA, T7432XD, T7432XS, T7492XA, T7492XD, T7492XS, T7602XA, T7602XD, T7602XS, T7612XA, T7612XS, T7612XD, T7622XA, T7622XD, T7622XS, T7632XA, T7632XD, T7632XS, T7692XA, T7692XD, T7692XS, Z550, Z551,
Environmental	V8701, V8702, V8709, V8712, V8719, V1584, V1586, V1589, V8732, V7181, Z77021, V8731, V1541, V1542, V1549, V6111, V6142, Z77090, Z77098, Z77110, Z77111, Z77118, Z77122, Z77128, Z77123, Z77010, Z77012, Z77018, Z77121, 9840, 9841, 99580, 99581, 99582, 99583, 99584, 99585, 9848, 9849, E9600, E9601, E961, E9620, E9621, E9622, E9629, E963, E964, E9650, E9651, E9652, E9653, E9654, E9655, E9656, E9658, E9659, E966, E9680, E9681,	O9A311, O9A312, O9A313, O9A319, O9A32, O9A33, O9A411, O9A412, O9A413, O9A419, O9A42, O9A43, O9A511, O9A512, O9A513, O9A519, O9A52, O9A53, T7491XA, T7491XD, T7491XS, T7431XA, T7431XD, T7431XS, T7421XA, T7421XD, T7421XS, T7411XA, T7411XD, T7411XS, T7401XA, T7401XD, T7401XS, T7601XA, T7601XD, T7601XS, T7611XA, T7611XD, T7611XS, T7621XA, T7621XD, T7621XS, T7631XA, T7631XD, T7631XS, T7691XA, T7691XD, T560X1A, T560X1D, T560X1S, T560X2A, T560X2D, T560X2S, T560X3A, T560X3D, T560X3S, T560X4A, T560X4D, T560X4S,

© 2023 Agarwal AR et al. *JAMA Network Open.*

	E9682, E9683, E9684, E9685, E9686, E9687, E9688, E9689, E969, E8615, E8660,	T7691XS, Z654, Z655, Z91410, Z91411, Z91412, Z91419, Z9142, Z9149, Z77011, Z77120, Z6911, Z6379, M1A10X0, M1A10X1,
Social	V625, V603, V604, V6101, V6102, V6103, V6104, V6105, V6106, V6107, V6108, V6109, V613, V6141, V6149, V618, V619, V624, V6281, V6282, V6289, V629, V692, V693, V694, V698, V699, V695	Z603, Z605, Z608, Z609, Z600, Z602, Z604, Z650, Z651, Z652, Z653, Z658, Z659, Z6331, Z6332, Z634, Z635, Z636, Z6371, Z6372, Z6379, Z638, Z639, Z630, Z631, Z732, Z733, Z7389, Z72810, Z72811, Z72820, Z72821, Z7289, Z73810, Z73811, Z73812, Z73819, Z739, F439
Healthcare	V638, V639	Z749, Z748, Z753, Z754, Z758, Z759

© 2023 Agarwal AR et al. *JAMA Network Open.*
