# Supplementary material for: Assessment of Use of ICD-9 and ICD-10 Codes for Social Determinants of Health in the US, 2011-2021
Source: JAMA Netw Open. 2023 May 9;6(5):e2312538. doi: 10.1001/jamanetworkopen.2023.12538 (PMC10170331; doi:10.1001/jamanetworkopen.2023.12538)
Supplement: Supplement 2. — Data Sharing Statement [file jamanetwopen-e2312538-s002.pdf]

Data Sharing Statement

Agarwal. Assessment of Use of ICD-9 and ICD-10 Codes for Social Determinants of Health in the US, 2011-2021. *JAMA Netw Open*. Published May 09, 2023.
doi:10.1001/jamanetworkopen.2023.12538

Data
Data available: No

Additional Information
Explanation for why data not available: The PearlDiver database only permits analysis of the data in its interface and does not permit the dissemination of patient information to users.
